# Experiments in Collaboration: Interdisciplinary Graduate Education in Science and Justice

**DOI:** 10.1371/journal.pbio.1001619

**Published:** 2013-07-30

**Authors:** Science & Justice Research Center (Collaborations Group)

**Affiliations:** University of California, Santa Cruz, Santa Cruz, California, United States of America; King's College London, United Kingdom

## Abstract

The Science & Justice Training Program at UCSC has developed an innovative approach to ethics education for scientists and engineers that emphasizes interdisciplinary and collaborative research into shared objects and concerns.

*This Community Page is part of the Public Engagement in Science series*

Over the past two decades, policy changes at the national level have created an increased focus on science-society relations. An example in the United States has been a subtle but significant shift in the foundational principles of the National Science Foundation (NSF): rather than assume societal benefits directly flow from support of science and engineering, the NSF now explicitly seeks to create knowledge that benefits society [1]–[4]. To achieve this goal, the agency moved in 1997 to adopt the Broader Impacts Criterion (BIC) to review grant proposals [5],[6]. Similarly, the 2007 America COMPETES Act increased ethics education requirements for graduate students and postdoctoral fellows without specifying content [7]–[10]. While these policy changes require scientists and engineers to practice science and engineering in new ways that engage “the public” and benefit “society,” few institutions provide physical spaces for cross-disciplinary contact and intellectual space for figuring out how practically to achieve these ends [10]–[13]. The spaces that do exist tend to focus on meeting relatively narrow and instrumental ends—teaching professional conduct and making sure mandated ethics courses are offered—rather than doing the more fundamental work of discerning the specific ways in which science and engineering research connect to societal issues and public concerns.

Within these new policies, however, we note an unexpected and underexploited benefit: where there is a mandate with little guidance, there is also an opportunity to innovate. We offer the University of California Santa Cruz (UCSC) Science & Justice Training Program (SJTP) as one example of the kind of space that is made possible by the current policy focus on creating closer relationships between science and engineering and the people they intend to serve. The SJTP has taken an innovative approach that: (1) emerges from specific research practices; and (2) expands the set of considerations that qualify as scientific responsibility [15]. In this Community Page, we lay out the main components of this approach: creating legitimate institutional space where the links between science and engineering and questions of ethics and justice might be explored; encouraging students to “slow down” to investigate these questions on the ground; and supporting collaborations that arise organically from common concerns.

## Creating Legitimate Institutional Space

Funded through an NSF grant awarded to UCSC in 2010, the SJTP is a graduate-level research and education program that trains science and engineering students alongside students of social science, arts, and humanities to respond to the ethical and social justice questions that arise in their research. Rather than treating justice as a concern to be tacked onto an already formed research project, SJTP graduate fellows are provided with fellowship funding and faculty mentorship that supports them to explore questions of ethics and justice as they arise in their research. They enroll in two seminars, one that emphasizes different models and approaches to the science/society interface, and a second that introduces them to interdisciplinary methods they can use in their own projects. The SJTP encourages collaboration among graduate fellows, faculty, and research staff from across the University's academic divisions as well as those outside the University with interests in the student's research area. Located under the auspices of the Science & Justice Research Center (http://scijust.ucsc.edu), the SJTP is synergistic with the Center's other efforts: the Science and Justice Working Group, in which faculty, students, and members of the public gather to address problems and issues of common concern, and monthly “Cocktail Hours” during which fellows can discuss their progress and challenges as they develop their SJTP projects. The Center itself also provides physical space conducive to these interactions.

## Foregrounding Justice

The space, funding, and institutional recognition of the program give fellows the opportunity to reorient their research questions, methodologies, and goals around questions of science and justice. Fellows receive institutional support for projects that might be more difficult to fit into a traditional PhD program. For example, two fellows are part of a physics laboratory working on developing solar greenhouse technology for industrial-scale agricultural operations. The luminescent greenhouse windows contain strips of solar cells that allow photosynthetically active radiation to pass through, while absorbing and converting other wavelengths to electrical energy. Using these luminescent panels, a farmer could produce the energy needed to run the infrastructure of the greenhouse (e.g., fans and electronic sensors and controls). Rather than having the technology solely target industrial agricultural outfits, these fellows planned to develop the technology for concurrent use by small-scale organic farmers. Using skills developed in the SJTP's research methods seminar, they interviewed small-scale organic farmers to explore possibilities for transferring the technology to them for their use. They found, however, that these farmers had deliberately avoided high-tech approaches and were thus not motivated by the solar cell technology the way that the fellows originally envisioned. The SJTP fellows then reconsidered which publics might benefit from the project, including educators. The greenhouse project will be used by the fellows as an educational tool for students to learn about sustainable agriculture and nutrition as well as properties of light and color under the luminescent solar concentrating roof.

This iterative process allows SJTP fellows to think about how different publics relate to new science and technology on the ground, and to adjust their expectations and projects to the practicalities of their collaborations. This approach differs from that usually taken when addressing the BIC of NSF grants. In many instances, when researchers write the section of their grant on the broader impacts of the research, they assume rather than investigate who makes up the publics of their research, and what they want from science and technology [2],[5],[9],[11]. By investing time into forging interdisciplinary skills and intellectual relationships, the SJTP creates room for researchers to test and reformulate their ideas about their project's connections to public constituencies and their values. What counts as a “broader impact” and a contribution to a more just world is not known in advance, but can emerge through this process of collaboration and experimentation [13],[16]–[19]. While this effort can be hard work, with frictions and dead ends along the way, the results are much more satisfying and effective. Helping students to learn how to respond to complex and changing contexts prepares them to work responsively and responsibly, as well as to take part in a more open approach to engagement that the previous comment in this series argued is ultimately more productive [20].

## Slow Science

One central way that SJTP supports this new approach is by creating a space for fellows to “slow down” to explore different possibilities for developing their projects. SJTP's ‘slow science’ responds to the pressure on academics to “publish or perish,” to move quickly through projects, and to be efficient and cost effective [21]–[23]. While efficiency will continue to be an important value in responding quickly to pressing social and scientific concerns, speed must be moderated by attention to issues of justice at every level of the research project: the initial framing of research questions, the methodologies used, the analysis of results, and the ongoing attention to the public implications of the research project [24]. The working premise is that the ethical and social justice issues cannot be known in advance but must be explored in each project individually; students learn by doing. SJTP offers fellows opportunities to try things out that might not work, labor through frustrations, and feel the freedom to do uncertain and experimental work outside of the “fast-track” of a structured PhD program. At the same time, the program is structured to support students in continuing to progress with their doctoral research projects. The directors purposely avoid adding too heavily to their significant research commitments and responsibilities while encouraging them to use SJTP activities for career building.

Slowing down the research process also allows fellows to connect with public concerns and build stronger ties with specific communities. For example, one fellow working on governmental public participation projects in environmental remediation heard citizen participants express the fear that science is being “fast tracked,” with projects rushed through without proper citizen evaluation or environmental review. Despite the implementation of several federal initiatives that aim to involve communities earlier and more effectively in the remediation process, many citizens participating in these programs stated that they believed they had limited influence on decision-making. As a response, the fellow designed her SJTP project to produce a policy report written collaboratively with key informants from the public that addresses the institutional barriers that may prevent meaningful citizen participation.

## Collaboration

A guiding principle of the SJTP is that no one person or discipline has the expertise to determine the conditions of scientific responsibility; thus, collaboration across traditionally distinct disciplines and realms of expertise is imperative. The goal of this collaboration is not to turn scientists into social scientists or humanities scholars or vice versa. Rather, it is to create opportunities for graduate students and other SJTP members to gather around common objects and concerns (e.g., a greenhouse, climate change, or the use of racial categories in biomedical research). The program has drawn on a number of theories to develop these practices of gathering, but common to each is the commitment that research practices that are more porous to other disciplinary expertise are both more empirically rigorous and ethically responsive [25]–[33].

These gatherings also help participants to reflect on the conventions of their own disciplines. For example, the fellows working on the solar greenhouse discovered that expectations of their program, the resources of their department, and established funding structures limited the questions they could ask in their dissertation research. Graduate education typically places heavy emphasis on transforming research into outputs easily recognized within disciplines, which can crowd out opportunities for innovating new relationships between disciplines [14],[34]. The SJTP gave them space and time to explore new questions, new methods, and new forms of collaborative inquiry that have opened up new research and teaching collaborations, making them stronger candidates for careers both inside and outside of the university.

The experimental, often informal feel of the Cocktail Hours (see Box 1) allows fellows to ask questions and have conversations that might not occur in more traditionally structured venues like laboratory meetings. They provide an atmosphere in which participants feel comfortable trying out only partially developed ideas, admitting to being uncertain or exploring research paths that do not traditionally fit into their home disciplines. These ongoing discussions break down barriers to communication and collaboration and create more nuanced understandings of science and publics that are more promising for building projects that address the ethical and political dimensions of science and technology.

**Box 1. Science and Justice Training Program in Practice: Conviviality Comes First**

Interdisciplinary research falters if it does not grow organically out of genuinely held mutual interests and concerns. Fostering interdisciplinary research requires creating spaces where mutual interests and concerns can be discovered without any rigid commitment to what the objects of research ought to be. To accomplish this goal, we have strived to build spaces that are inviting, friendly, and open to unexpected inspirations and connections. Work from our artists-in-residence hangs on the walls; the redwood forest outside of our Center windows provides a sense of possibility.

**Cocktail Hours**: Bi-weekly, late-afternoon informal meetings feature presentations of graduate student research, films, or seminars from visiting scholars. These are low-pressure, low-stakes conversations intended to be both fun and intellectually refreshing. Because the primary purpose of these meetings is to build familiarity and trust amongst our graduate students and closely involved faculty, they typically are not advertised on campus-wide listservs. Food and drink are provided by the Center.

**Common Space**: Establishing a permanent space on campus was important to the growth of our graduate program. Because space is often at a premium, and establishing access to a room can be arduous for graduate students, having a common space not associated with a department and that graduate students have access to at any time allows casual or spontaneous organization of collaborative work and informal seminars.

**Working Group Meetings**: Bi-weekly working group meetings are attended by people from all across our university and the local community. They depart from a typical colloquia format by inviting presenters to avoid giving polished PowerPoint presentations and to instead share a question or problem that would benefit from cross-disciplinary discussion. Presenters are encouraged to avoid jargon and the audience is encouraged to ask about unfamiliar terms and concepts. Attendees typically break at a midway point to share snacks and socialize, before resuming for a question and answer period. The social time allows members to meet or catch-up and to discuss the topic at hand in a more informal setting.

**Interdisciplinary Seminars**: The program does not endeavor to un- or re-discipline students because their projects need to be legible to their own disciplines. Rather, the aim is to create a space where common language and methods can be innovated together. Students are asked to bring in exemplars or elements of their objects of study as well as to learn to read these objects with different lenses that bring into focus the places where their objects of study meet questions of ethics and justice. Many of the methods used to cultivate these readings are inspired by the theories and methods of science and society scholars, but are heavily adapted in practice so that they are legible across disciplinary lines. Courses are cross-listed in each of the university's academic divisions, ensuring that students will receive credit hours that are recognized within their home departments.

As one example, the SJTP gave a fellow working on mathematical models of fishery conservation the chance to experiment with a project to address the lack of ecological data about subsistence fishing in an area of Sierra Leone. She used interviews, a method explored in the training program, to begin to develop localized knowledge about fish stocks. While SJTP initially helped by introducing her to new methods and providing institutional support to proceed with this project, the Cocktail Hours also gave her a space to return to after her time in the field where she could reflect on how a lack of mutual trust between her and interview subjects can hamper responsible data collection. She concluded that what at first appeared a problem (lack of data) that could be solved through research was in fact entangled with historical and political issues that required a broader perspective to navigate. SJTP has created this interdisciplinary space that enables critical reformulations of what collaborative practices with community members entails.

Sometimes this reformulation has led to the hard work of critically examining our own well-intentioned approaches to working across divides. SJTP's Climate Cluster, a subset of fellows who have backgrounds in civil engineering, politics, and environmental studies, sought to create public conversations that brought questions about social and environmental justice together with questions about the current state of knowledge in climate science. While sharing this common goal, when it came to planning and preparing for the interdisciplinary series, members of the Cluster found that they held different assumptions about which types of experts to invite and how to engage them. The Climate Cluster experience demonstrated that arriving at a shared vision for the events required practices of listening and negotiating different disciplinary approaches to constituting substantive public discussion. The procedures for communication and collaboration across disciplines, perspectives, and interests that they developed during these discussions laid the groundwork for future collaborative projects [35]. Using the panel series as a springboard, the Climate Cluster fellows successfully mobilized new collaborative groups comprised of academic and public and private sector actors. This led, for example, to the formation of a new Interdisciplinary Development Working Group that gathered at the Research Center for a day-long workshop entitled “Rethinking Development in Light of Climate Change,” with participants from academia and the public municipal sphere.

These conditions of collaboration highlight some important aspects of how we understand ‘justice,’ and why we conjoined it with ‘science.’ The concept of justice contains a multitude of meanings. It is commonly assumed that we are either referring to the judicial or legal meaning, or only addressing traditional social justice concerns of rectifying longstanding structural inequalities. While both of these elements are certainly important to the program, the primary concern is to create the conditions for people with expertise in multiple disciplines to gather around, and in that process create a notion of the common good. Importantly, this common good is not built around a particular theory of justice. Rather, the goal has been to generate knowledge practices that are empirically robust, modest in scope, and responsive to the conditions of a just society that we envision together. In SJTP, justice operates in an aspirational sense to inspire the constructive modes of engagement across intellectual boundaries that make this knowledge possible.

## Conclusion

A common theme in the experiences of the SJTP fellows has been the realization that there are many experts and publics with conflicting expectations about what counts as scientific responsibility and constructive public engagement. The SJTP's creation of legitimate institutional space that allows students to slow down and creatively address these differences fosters responsible science and engineering from the bottom up. It demonstrates that one aspect of creating successful engagements across so-called public and expert domains is to train experts who are able to respond to these differences and thus foster more open forms of collaboration. In a world increasingly shaped by science and technology, the SJTP aims to offer one pathway for science and engineering to connect to social issues and public concerns in a more practical, substantive, and thoughtful way.

## Abbreviations

BIC: Broader Impacts Criterion
NSF: National Science Foundation
SJTP: Science & Justice Training Program

## References

[1] National Science Foundation (1995) NSF in a Changing World: The National Science Foundation's Strategic Plan (NSF 95-24). Available: http://www.nsf.gov/nsf/nsfpubs/straplan/contents.htm. Accessed March 9, 2013.

[2] Holbrook JB (2005) Assessing the science–society relation: The case of the US National Science Foundation's second merit review criterion. Technology in Society 27: 437–451 10.1016/j.techsoc.2005.08.001.

[3] Roberts MR (2009) Realizing societal benefit from academic research: analysis of the national science foundation's broader impacts criterion. Social Epistemology 23: 199–219 10.1080/02691720903364035

[4] Frodeman R, Holbrook JB (2011) NSF's struggle to articulate relevance. Science 333: 157. 21737723 10.1126/science.333.6039.157

[5] Mervis J (2011) Beyond the data. Science 334: 169–171. 21998363 10.1126/science.334.6053.169

[6] Lok C (2010) Science funding: Science for the masses. Nature 465: 416. 20505707 10.1038/465416a

[7] America COMPETE Act 2007 H.R. 2272 Section 7009 Responsible Conduct of Research. Signed 10 May 2007. Available: http://www.govtrack.us/congress/bills/110/hr2272. Accessed March 14, 2013.

[8] Tuana N (2010) Leading with ethics, aiming for policy: new opportunities for philosophy of science. Synthese 177: 471–492 10.1007/s11229-010-9793-4

[9] Schienke EW, Tuana N, Brown DA, Davis KJ, Keller K, et al (2009) The role of the National Science Foundation broader impacts criterion in enhancing research ethics pedagogy. Social Epistemology 23: 317–336 10.1080/02691720903364282

[10] Although the content of ethics education is not mandated, as part of the broader effort to articulate science to social ends, attempts have been made to produce and disseminate texts and curricula to assist ethics education. For a sampling of these efforts, see the National Ethics Collaborative Online Research Environment (CORE), including a collection of case studies and literature, Available: http://nationalethicscenter.org. Accessed March 14, 2013.

[11] Tuana N (2012) Embedding philosophers in the practices of science: bringing humanities to the sciences. Synthese 10.1007/s11229-012-0171-2

[12] Schneider J, Lucena J, Leydens (2009) Engineering to help: the value of critique in engineering service. IEEE Technology and Society Magazine 10.1109/MTS.2009.935008

[13] Reardon J (2013) On the Emergence of Science and Justice. Sci Technol Human Values 38: 176–200 10.1177/0162243912473161

[14] Rhoten D (2004) Interdisciplinary research: Trend or transition. Items Issues 5: 6–11.

[15] Our approach draws heavily from academic fields, such as Feminist Science Studies and Science and Technology Studies, that over the past three decades have developed a robust understanding of science-society relationships.

[16] Gibson-Graham K (2011) A feminist project of belonging for the Anthropocene. Gend Place Cult 18: 1–21.

[17] Mamo L, Fishman R (2013) Why justice?: introduction to the special issue on entanglements of science, ethics, and justice. Sci Technol Human Values 38: 159–175.

[18] Star S (2010) This is not a boundary object: reflections on the origin of a concept. Sci Technol Human Values 35: 601–617.

[19] Choy TK, Faier L, Hathaway MJ, Inoue M, Satsuaka S, Tsing A (2009) A new form of collaboration in cultural anthropology: Matsutake worlds. American Ethnologist 36: 380–403.

[20] Stirling A (2010) Opening up the politics of knowledge and power in bioscience. PLoS Biol 10 1: e1001233 doi:10.1371/journal.pbio.1001233 10.1371/journal.pbio.1001233 PMC3250508 22235193

[21] Müller R (2012) Collaborating in life science research groups: the question of authorship. Higher Education Policy 25: 289–311.

[22] Lutz JF (2012) Slow science. Nat Chem 4: 588–589. 22824878 10.1038/nchem.1415

[23] McCabe D (2012) The Slow Science Movement. University Affairs/Affaires universitaires 5 Dec 2011. Available: http://www.universityaffairs.ca/the-slow-science-movement.aspx. Accessed March 9, 2013

[24] Stengers I (2011) “Another Science is Possible!” A Plea for Slow Science. Inaugural Lecture for Willy Calewaert Chair. 13 December 2011. Available: http://threerottenpotatoes.files.wordpress.com/2011/06/stengers2011_pleaslowscience.pdf. Accessed March 9, 2013.

[25] Haraway DJ (1991) Simians, cyborgs and women: the reinvention of nature. London: Free Association.

[26] Haraway DJ (1997) Modest-Witness@Second-Millennium.FemaleMan-Meets-OncoMouse: feminism and technoscience. New York: Psychology Press.

[27] Barad KM (2007) Meeting the Universe Halfway: Quantum Physics and The Entanglement of Matter And Meaning. Durham, NC: Duke University Press.

[28] Puig de la Bellacasa M (2011) Matters of care in technoscience: assembling neglected things. Social Studies of Science 41: 85–106. 21553641 10.1177/0306312710380301

[29] Latour B (2004) Why has critique run out of steam? From matters of fact to matters of concern. Critical Inquiry 30: 225–248.

[30] Latour B (2008) What is the style of matters of concern. Two lectures in empirical philosophy. Department of Philosophy of the University of Amsterdam, Amsterdam: Van Gorcum

[31] Star SL, Griesemer JR (1989) Institutional ecology,translations' and boundary objects: amateurs and professionals in Berkeley's Museum of Vertebrate Zoology, 1907–39. Social Studies of Science 19: 387–420.

[32] Star SL (1991) Power, technology, and the phenomenology of conventions: on being allergic to onions. In: Law J, editors. A Sociology of Monsters: Essays on Power, Technology, and the Domination. New York: Routledge.

[33] Reardon JR (2005). Race to the Finish: Identity and Governance in an Age of Genomics. Princeton, NJ: Princeton University Press.

[34] Golde CM, Gallagher HA (1999) The challenges of conducting interdisciplinary research in traditional doctoral programs. Ecosystems 2: 281–285.

[35] Focusing on communication styles has also been key to the success of the Science and Justice Working Group meetings. In those meetings, participants are encouraged to ask others to explain specialized language. At times, the ‘red flag’ method is used to encourage members to place use of words and phrases they find troubling on the board. The goal is not to resolve the discomfort, but to at least recognize it.

